# Follicular expression of follicle stimulating hormone receptor variants in the ewe

**DOI:** 10.1186/1477-7827-11-113

**Published:** 2013-12-14

**Authors:** Rachael R Sullivan, Brian R Faris, Douglas Eborn, David M Grieger, Ada G Cino-Ozuna, Timothy G Rozell

**Affiliations:** 1 Biosecurity Research Institute, Kansas State University, Manhattan, KS 66506, USA; 2 Department of Animal Sciences and Industry, Kansas State University, Manhattan, KS 66506, USA; 3 Roman L. Hruska U.S. Meat Animal Research Center, Clay Center, NE, USA; 4 Department of Diagnostic Medicine/Pathobiology, College of Veterinary Medicine, Kansas State University, Manhattan, KS 66506, USA

**Keywords:** FSH receptor, Follicle development, Ewe, CIDR, Alternate splicing

## Abstract

**Background:**

Several alternatively-spliced mRNA transcripts of the follicle stimulating hormone receptor (*FSHR*) have been identified in sheep, including FSHR-1 (G protein-coupled form), FSHR-2 (dominant negative form), and FSHR-3 (growth factor type-1 form). Our objective was to determine which of these variants is predominantly expressed in follicles collected from ewes at various times after estrus.

**Methods:**

Suffolk-cross ewes (n = 8) were allowed to come into estrus naturally and were euthanized 24 (n = 3), 36 (n = 3), or 48 (n = 2) hours after the onset of estrus. All visible follicles were measured, aspirated and pooled according to follicular diameter: small (<= 2.0 mm), medium (2.1-4.0 mm), large (4.1-6.0 mm), and preovulatory (> = 6.1 mm). Aspirated cells were separated from follicular fluid by centrifugation. Total RNA was extracted from cell pellets and reverse transcribed. The resulting cDNA was subjected to qPCR, using primer sets designed to amplify each variant specifically. Gene expression was normalized to that of beta–actin within samples, and compared by analysis of variance with the level of significant differences set at p < .05.

**Results:**

Relative expression of FSHR-3 exceeded that of both FSHR-1 and FSHR-2 in medium follicles, and tended to be higher in small follicles (p = .09) regardless of time after onset of estrus, and thus results from different time points were pooled. Expression of FSHR-3 was greater than that of FSHR-2 and luteinizing hormone receptor (LHR) in small and medium follicles. Expression of LHR was greatest in preovulatory follicles.

**Conclusions:**

These experiments show that in addition to the well characterized G protein-coupled form of the FSHR, alternatively spliced variants of the FSHR may participate in follicular dynamics during follicular waves of the sheep estrous cycle. Furthermore, these results indicate that an “alternatively” spliced form of the FSHR (FSHR-3) is the predominant form of the FSHR in the sheep.

## Background

For successful reproduction to occur, follicles must develop through several stages within the ovary. Antral follicle growth is regulated largely by follicle stimulating hormone (FSH) and luteinizing hormone (LH) from the pituitary. In order for FSH and LH to exert their effects, the appropriate receptors must be present on follicular cells at the correct time. At least one form of the follicle stimulating hormone receptor (FSHR) is detectable soon after follicle formation in sheep [1]. Several alternatively spliced FSHR mRNA variants have been identified in sheep [1-4]. Each splice variant has a unique exon structure that may dictate receptor coupling to signaling molecules.

Messenger RNA for the G protein-coupled form of the FSHR (FSHR-1) was first sequenced and described in the rat in 1990 [5] and in the sheep in 1993 [2]. Sheep FSHR-1 mRNA is 2431 base pairs (bp) in length, and consists of 10 exons. Once translated, FSHR-1 is capable of activating several intracellular signaling pathways, but cAMP/PKA is the most commonly described pathway (as reviewed by [6]). The FSHR-1 form is important for granulosa cell (GC) differentiation and hormone production, as well as GC proliferation (as reviewed by [7]).

Other identified splice variants include FSHR-2 (dominant negative receptor) and FSHR-3 (growth factor type-1 receptor). The exon structure of FSHR-2 is similar to that of FSHR-1, except that FSHR-2 has a truncated exon 10 spliced to exon 11 [4]. The truncation in exon 10 is thought to affect receptor signaling, potentially by altering the intracellular portions of the receptor [4]. Although FSHR-2 has been shown to bind FSH with high affinity, downstream signaling did not occur [8]. In fact, when FSHR-2 and FSHR-1 were co-transfected into HEK 293 cells and treated with FSH, only a very small increase in intracellular cAMP was detectable, in contrast to the large increase caused by cells expressing FSHR-1 alone. Thus, FSHR-2 appeared to attenuate the actions of FSHR-1, leading the authors to conclude that FSHR-2 acts as a dominant negative form of the FSHR [8].

Exons 1–8 encoding FSHR-3 are identical to that of FSHR-1; however, FSHR-3 lacks exons 9 and 10, and the first 8 exons are spliced directly to exon 11 [3,9]. In contrast to FSHR-1, FSHR-3 may act in a cAMP-independent fashion. When stimulated by FSH, it has been shown to activate a mitogen-activated protein kinase (MAPK) pathway, specifically the extracellular-regulated kinase (ERK) signaling cascade [10]. The ERK cascade is involved in cell proliferation, is regulated by Ras and is regulated by a Ca^2+^ dependent process. When Touyz et al. [11] transfected HEK 293 cells with FSHR-3, treatment with FSH resulted in a dramatic increase in Ca^2±^. The significance of these findings relates to the increases in cell proliferation observed shortly after calcium influx and activation of the ERK signaling cascade [11]. These studies provide possible evidence that FSH can stimulate granulosa cell proliferation directly through FSHR-3.

In the cow, FSHR transcripts containing exon 11 (FSHR-2 or FSHR-3) were more highly expressed in cohort than in dominant follicles [12]. However, FSHR transcripts containing exon 10 (FSHR-1) were more highly expressed in cohort and dominant follicles than in subordinate follicles during the estrous cycle [12]. Based on those findings we postulated that alternative FSHR transcripts, in addition to the well characterized FSHR-1 form, may be expressed differently in differently sized follicles under normal physiological conditions in the ewe.

## Methods

### Animals and sample collection

All animal procedures were approved by the Kansas State University Animal Care and Use Committee (IACUC protocol #2735). Ewes were group housed at the KSU Sheep Unit in a south-facing lean-to barn and were exposed to natural lighting and temperature (39° 12′ 22″ N, 96° 35′ 12″ W). Ewes had free access to water and were fed a maintenance diet of grass hay and 2 pounds/head/day of a grain supplement (87% DM; 14% CP; 85% TDN). Ewes were transported from the KSU Sheep Unit to the KSU Diagnostic Lab for euthanasia. When ewes were loaded onto the trailer, blood samples were collected via jugular venipuncture and placed on ice until refrigeration at 4°C for 24 hours. Serum was separated by centrifugation at 1500×g for 20 minutes at 4°C. Serum was decanted into a 5 ml polypropylene tube, capped and stored at −20°C until assay. Time from loading ewes to euthanasia was less than 1 hour. Ewes were euthanized by intravenous overdose (90 mg/kg) of sodium pentobarbital.

Eight mature cycling (2–8 years of age) Suffolk-cross ewes were allowed to come into estrus naturally. Estrous cycle length was monitored using a HeatWatch® detection system (Denver, CO) and two vasectomized rams through 3 consecutive cycles before harvesting ovaries at the end of the breeding season in late January through early February. Onset of estrus was defined as the beginning of the period that a ewe was receptive to mounting by the ram. After a HeatWatch® transmitter was activated by a ram, estrous behavior was visually confirmed. Ewes were euthanized 24 (n = 3), 36 (n = 3), or 48 (n = 2) hours after onset of estrus. Ovaries were harvested and placed on ice before transport to the laboratory for evaluation. Left and right ovaries of each ewe were kept separate until photographed and all ovarian structures were measured (to the nearest 0.5 mm) and recorded. Ovarian structures were measured with a transparent ruler from the external surface of the ovary. All visible follicles were aspirated using 1 cc syringes and 20 gauge needles. Each follicle was aspirated such that the needle was inserted, follicular fluid was pulled into the syringe and then gently pushed back into the same follicle three times before removing the needle and moving to the next follicle. A new syringe and needle were used for each ewe and each follicle size class. Aspirates were pooled in 1.5 ml microcentrifuge tubes according to follicular diameter: small (≤ 2.0 mm), medium (2.1-4.0 mm), large (4.1-6.0 mm) and preovulatory (≥ 6.1 mm). Follicular fluid and aspirated cells (should be primarily granulosa cells, but the presence of thecal cells within the pellet was not determined) were separated by centrifugation at room temperature for two minutes at 2300×g. Follicular fluid was removed and stored at −20°C for estrogen and progesterone assays.

Cell pellets were resuspended in 500 μl ice-cold Dulbecco’s Phosphate Buffered Saline (PBS; Invitrogen, Carlsbad, CA) and centrifuged at room temperature for one minute at 2300×g. The PBS was aspirated and discarded and the cell pellets were resuspended in 1 ml TRIzol® reagent (Life Technologies, Grand Island, NY) and frozen at −20°C until all samples were collected. Time between ovary harvest and placing cells in TRIzol® reagent did not exceed 2 hours.

### RNA isolation and DNase treatment

RNA was isolated from samples using TRIzol® reagent according to the manufacturer’s instructions. The RNA pellet was washed with 75% EtOH and resuspended in 15 μl preheated (65°C) nuclease-free water (Ambion®, Austin, TX). All RNA extracts were treated with TURBO DNA-*free™* (DNase; Ambion®) according to the manufacturer’s instructions to remove any genomic or other cellular DNA and stored at −20°C.

### Reverse transcription – polymerase chain reaction

Total RNA (100 ng) was used as the starting template for RT-PCR. Some samples with low (≤ 50 ng/μl) concentrations of total RNA initially failed to produce consistent real-time PCR products. Total RNA from those samples was then reverse-transcribed using 200 or 300 ng total RNA to ultimately ensure adequate template for real time PCR. Total RNA was reverse-transcribed using SuperScript™ III First-Strand Synthesis SuperMix (Invitrogen), according to the manufacturer’s instructions. Conditions were as follows for reverse transcription: 25°C hold for 25 minutes, 42°C for 50 minutes, 85°C for 5 minutes. At the end of the reverse-transcription reaction, samples were placed on ice for at least 1 minute. To remove template RNA, 1 μl RNase H was added to each sample and samples were incubated at 37°C for 20 minutes. Reactions were stored at −20°C until used for qPCR.

### Real-time PCR

**Figure 1:**
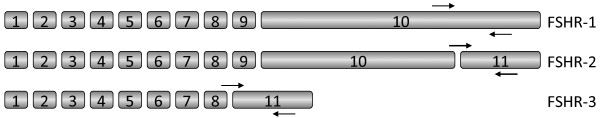
**Location of primers to specifically amplify FSH receptor splice variants.** Primer pairs used for real time PCR were designed to result in amplicons unique to each specific form of the receptor as follows: FSHR-1—primer annealing sites within the unique region of exon 10 only found in this variant form of the receptor; FSHR-2—upstream primer annealing sites overlapping the exon 10 and 11 boundary only found in this variant; FSHR-3—upstream primer annealing sites overlapping the exon 8 and 11 boundary only found in this variant. Predicted sizes of amplicons were verified on an agarose gel (not shown), and melt curve analysis was performed to verify single products.

Real-time PCR was performed using an ABI 7500 Fast machine (Applied Biosystems, Foster City, CA) with the following parameters: 50°C for 2 min, 95°C for 2 min followed by 40 cycles of: 95°C for 15 sec, 60°C for 30 sec. Platinum® SYBR® Green qPCR SuperMix-UDG (Invitrogen™) was used at 10 μl per well; forward and reverse primers were used at 250 nM concentrations, cDNA template was diluted in 5 μl total volume using nuclease-free water, and nuclease-free water was used to bring the final reaction volume to 20 μl. Melt-curve analysis was performed on all samples after amplification, by heating products to 95°C and plotting the derivative of the reporter (fluorescence) and temperature at which products denature. All primers were designed using Primer Express® 3.0 software (Applied Biosystems) and synthesized by Applied Biosystems. Expression of β-actin was used as an internal standard. Primers (Table 1) used to detect FSHR-1, FSHR-2, FSHR-3 and LHR were designed to amplify each variant specifically (Figure 1), based on unique exon boundaries and were developed from submitted nucleotide sequences of each variant as referenced in Table 1.

**Table 1:** Description of primers used for quantitative real-time PCR

**Target**	**Accession no.**	**Primer name**	**Sequence 5’ to 3’**	**Length**	**Amplicon**
*β -Actin*	NM_001009784	oBeta-Actin For	GTCATCACCATCGGCAATGA	20	88
		oBeta-Actin Rev	CGTGAATGCCGCAGGATT	18	
*FSHR-1*	L07302	oExon10 For	CATTCACTGCCCACAACTTTCATC	24	84
		oExon10 Rev	TGAGTGTGTAATTGGAACCATTGGT	25	
*FSHR-2*	NM_001009289	oExon10/11 For	CAGGAACTTCCGCAGGGATT	20	72
		oExon10/11 Rev	TGATTGCAGATGAGCCCAACA	21	
*FSHR-3*	L12767	oExon8/11 For	CAGTAATTTGGAAGAACTGCCTAATG	26	80
		oExon8/11 Rev	AACAGTGCAGCAGTGGAGACA	21	
*LHR*	L36329	oLHR For	AGATTGCTAAGAAAATGGCAGTCCTCT	28	82
		oLHR Rev	GCAGCTGAGATGGCAAAGAAAGAGA	25	

Primer efficiencies ranged from 85 to 100%. Primer efficiencies under 95% were corrected to 100% so that comparisons between genes could be made.

### Radioimmunoassay

Duplicate aliquots (50 μl) of serum were assayed for progesterone concentration, as described and validated previously [13]. Sensitivity of the assay was 3.5 pg/tube and the mean intra- and inter-assay coefficients of variation (CV) were 4.75% and 4.55%, respectively. Duplicate aliquots (200 μl total volume) of follicular fluid were assayed without extraction for estradiol and progesterone, using previously described and validated assays [14,15]. Briefly, for estradiol evaluation, follicular fluid from small and medium-sized follicles was diluted 1:100 and follicular fluid from preovulatory follicles was diluted 1:500 prior to assay. Estradiol concentration was determined using a double antibody kit (Siemens Medical Solutions Diagnostics; Deerfield, IL). Sensitivity of the assay was 0.02 pg/tube and the mean intra-assay CV was 1.6%. Progesterone concentrations were determined using Coat-A-Count antibody coated tubes from Siemens Medical Solutions Diagnostics. For progesterone evaluation, follicular fluid from all follicle sizes was diluted 1:50. Sensitivity of the assay was 0.012 ng/tube and the mean intra-assay CV was 9.35%.

### Statistical analysis

Relative expression (2.0-∆Ct × 1000) values were used for statistical analysis of real-time PCR data. Data were analyzed with SAS 9.1 software (SAS Institute, Cary, NC) using Proc Mixed and least squared means. The experiment had a completely randomized design with a split-plot, with the split-plot having treatments in a 2 (follicle size) × 4 (gene) factorial. The ANOVA models included all treatment factor main effects and interactions; ewe was included as a random effect. Normality of residuals was tested and data transformed if necessary. The whole-plot treatment factor is hour and experimental unit is ewe. Gene expression in preovulatory follicles was analyzed separately because this size was only present in the 24-hour group. Untransformed data are presented as least squares means of relative expression ± SEM. After least squared means were calculated, they were back-transformed (by exponentially raising the transformed mean values). Confidence intervals for the transformed means were calculated so new standard errors could be assigned. Back-transformed data are presented as least squares means of relative expression, ± SEM.

## Results

**Figure 2:**
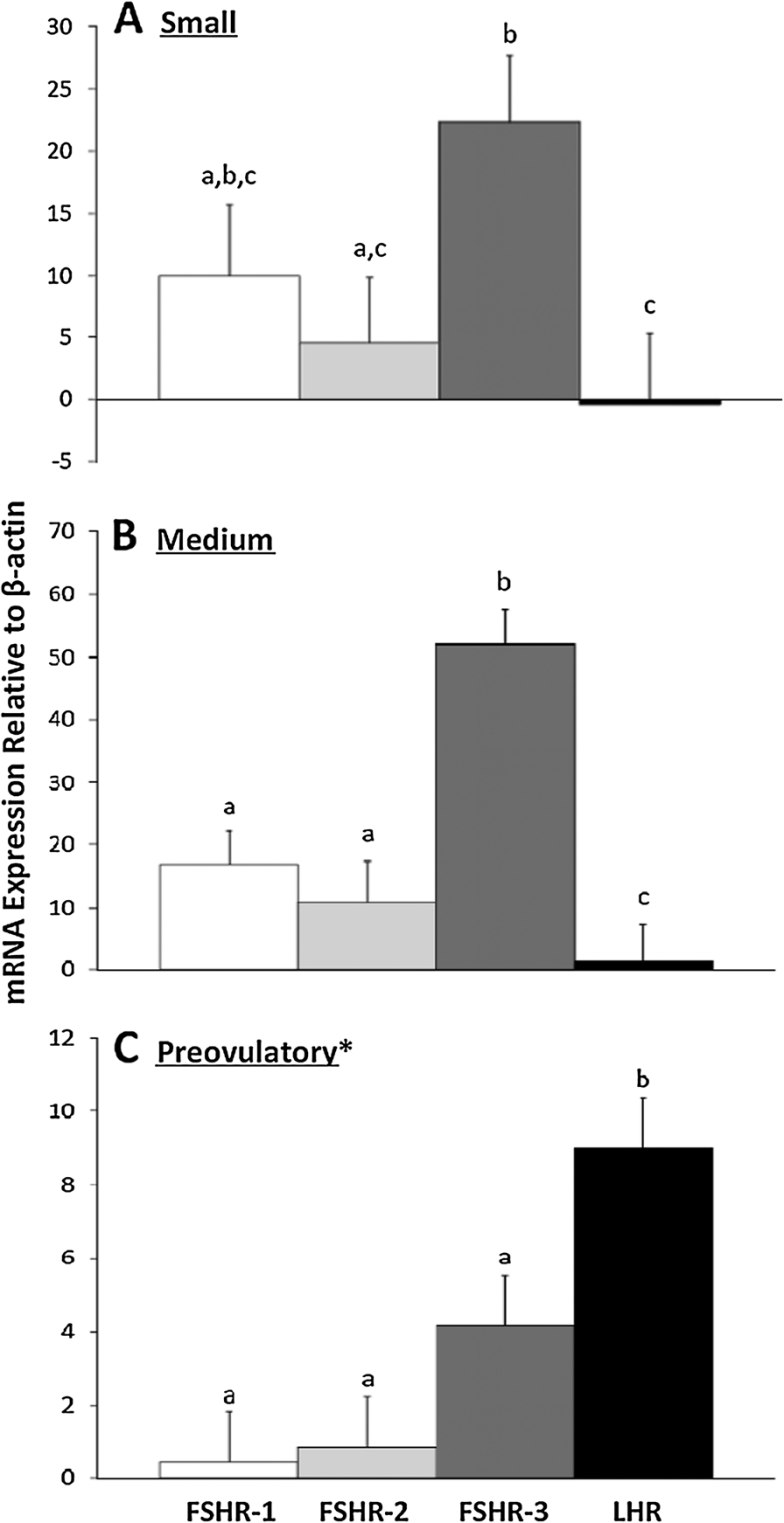
**Expression of gonadotropin receptors within different sized sheep follicles.** Cells were collected by centrifugation from follicular fluid aspirated from small (≤ 2.0 mm), medium (2.1-4.0 mm) and preovulatory (≥ 6.1 mm; *only found in the 24 hour group) follicles. Results from real time PCR are expressed as least squares means of expression relative to β-actin (2-∆Ct ×1000; arbitrary units) ± SEM. Bars within graphs with differing superscripts are different (p < .05). Expression of FSHR-1 (white bars), FSHR-2 (light gray bars), FSHR-3 (dark gray bars), and LHR (black bars) are compared within a follicle size class. Panel **A)** Relative expression of FSHR-3 was higher than FSHR-2 and LHR, and tended to be higher than FSHR-1 (*p = .09) in small follicles. Panel **B)** Relative expression of FSHR-3 was higher than other variants and LHR in medium follicles. Relative abundance of mRNA for FSHR-1 and FSHR-2 did not differ, but both were more highly expressed than LHR. Panel **C)** Relative expression of LHR was the highest in preovulatory follicles, while FSHR-1, FSHR-2, and FSHR-3 did not differ.

Ewes were euthanized at 24 (n = 3), 36 (n = 3) or 48 (n = 2) hours after onset of estrus. Expression of FSHR variant and LHR mRNA were not found to be different at the different time points (p > .05), and thus the following results are mean expression of genes from follicles collected within all time points. When comparing all FSHR variants and LHR within a follicle class size, FSHR-3 was more highly expressed than FSHR-1 in medium follicles (p < .05; Figure 2), and tended to be higher in small follicles (p = .09). Relative expression of FSHR-3 was higher than FSHR-2 in small and medium follicles (p < .05; Figure 2, panels A and B). In preovulatory follicles, LHR expression increased dramatically and was higher than all FSHR variants (p < .05); expression of FSHR variants was numerically lower than in small and medium follicles (Figure 2, panel C).

**Figure 3:**
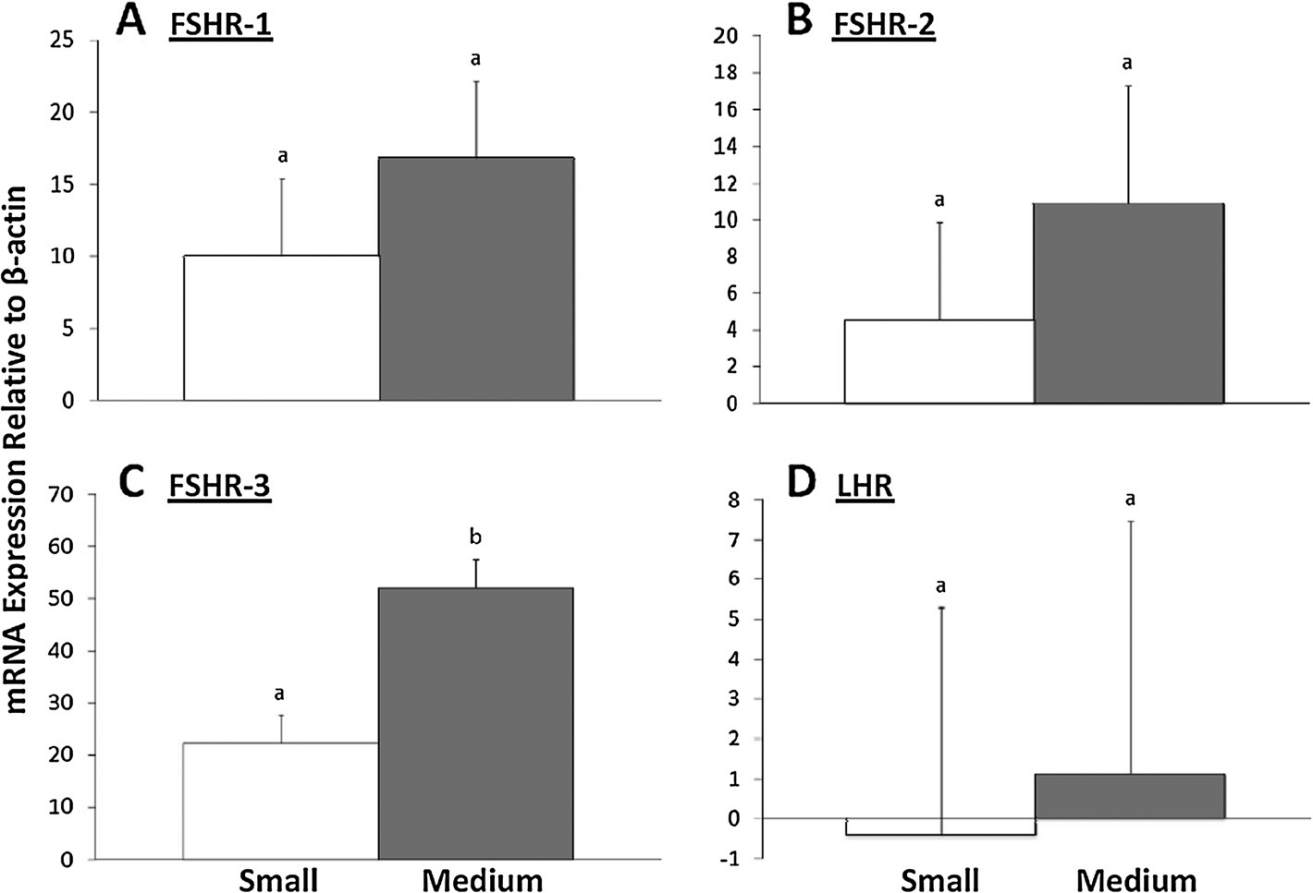
**Expression of gonadotropin receptors across different sized follicles.** Cells were collected by centrifugation from follicular fluid aspirated from small (≤ 2.0 mm; white bars) or medium (2.1-4.0 mm; gray bars) follicles. Results from real time PCR are expressed as least squares means of expression relative to β-actin (2-∆Ct ×1000; arbitrary units) ± SEM. Bars within graphs with differing superscripts are different (p < 0.05). Panels **A)**, **B)**, and **D)** Relative expression of FSHR-1, FSHR-2, and LHR did not significantly differ in small versus medium follicles. Panel **C)** Relative expression of FSHR-3 was higher in medium than small follicles. Overall, FSHR-3 expression was the only transcript that differed between small and medium follicles.

When comparing expression of each variant between small and medium follicles (Figure 3), neither FSHR-1 nor FSHR-2 was differentially expressed (p = 0.34 and 0.42, respectively); however, FSHR-3 expression was higher in medium than in small follicles (p < .05). Relative expression of LHR was essentially undetectable in small follicles and very low, on average, in medium follicles (p = .85).

**Figure 4:**
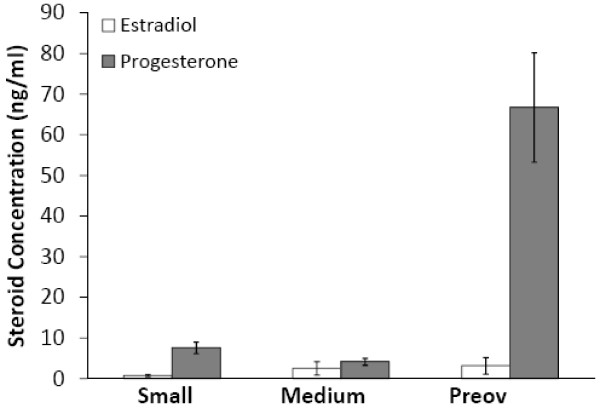
**Estradiol and progesterone concentrations in follicular fluid from different-sized sheep follicles.** Follicular fluid was pooled according to follicle diameter: small (≤ 2.0 mm), medium (2.1-4.0 mm) and preovulatory (preov; ≥ 6.1 mm). After cells were separated by centrifugation, duplicate aliquots (200 μl total volume) of follicular fluid were analyzed by RIA without extraction. Progesterone concentrations were consistently higher than estradiol in small, medium and preovulatory follicular fluid pools. Data are expressed as mean ± SEM.

Follicular fluid estrogen and progesterone concentrations were determined using RIA. Estradiol concentrations were consistently lower than progesterone (Figure 4); consequently, only one sample had an estradiol:progesterone ratio that exceeded 1.0. That sample, (Ewe 4, 48 hour; n = 3 medium follicles) had an E:P ratio of 2.0 and could therefore be considered a pool of healthy follicles, at least on average.

Estradiol concentrations from small follicle pools averaged 0.77 ng/ml and ranged from 0.07 ng/ml to 2.26 ng/ml. Estradiol concentrations for medium follicles averaged 2.53 ng/ml with a range from 0.05 ng/ml to 13.60 ng/ml). Estradiol in preovulatory follicles averaged 3.18 ng/ml. Preovulatory follicles were only present in the 24 hour group, with Ewe 12 (24 hour; n = 2 follicles) having the lowest (0.91 ng/ml) estradiol concentration and Ewe 16 (24 hour; n = 2 follicles) having the highest (7.28 ng/ml) concentration. Mean progesterone concentration in small follicles was 7.61 ng/ml and ranged from 1.61—10.97 ng/ml. Progesterone concentration in medium follicles averaged 4.18 ng/ml and ranged from 1.38—7.21 ng/ml. Preovulatory follicles had the highest mean progesterone concentration at 66.74 ng/ml, ranging from 43.70—90.29 ng/ml.

**Figure 5:**
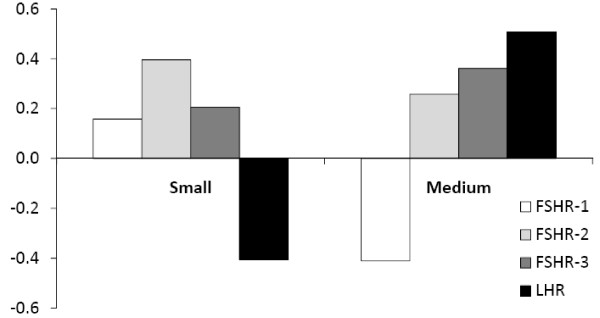
**Correlation between estradiol to progesterone ratio and gene expression in small and medium sheep follicles.** Follicular fluid was pooled according to follicle diameter: small (≤ 2.0 mm) and medium (2.1-4.0 mm). After cells were separated by centrifugation, duplicate aliquots (200 μl total volume) of follicular fluid were analyzed by RIA without extraction. Estradiol and progesterone concentrations were determined for each sample, and the ratio of estradiol to progesterone was calculated, then analyzed for correlation to FSHR or LHR gene expression. The FSHR-2 variant had the highest correlation (R = 0.4) to the E:P ratio compared to other *FSHR* variants. The E:P was inversely correlated to *LHR* expression in small follicles (R = −0.41), and FSHR-1 expression in medium follicles (R = −0.41). Data are expressed as correlation (R) values.

The E:P ratio was compared to relative gene expression of each transcript. Correlation values for for E:P ratio with gene expression are shown in Figure 5. Correlation between E:P and expression was the highest for FSHR-2 in small follicles (R = 0.4), although FSHR-2 was still the least expressed transcript. In medium follicles, the highest correlation was between E:P and LHR (R = 0.51).

Serum progesterone concentrations were evaluated from blood samples taken when ewes were loaded for transport. Serum progesterone values ranged from 0.35 - 0.67 ng/ml (Table 2).

**Table 2:** Average values for sample collection parameters in experiment 1 and 2

**Group**	**Time to euthanasia**	**Mean cycle length**	**Number of follicles**				**Luteal tissue (#)**			**Serum progesterone**
**Exp 1**	**(hours)**	**(days)**	**≤ 2 mm**	**2.1-4 mm**	**4.1-6 mm**	**≥ 6.1 mm**	**CH**	**CL**	**CA**	**(ng/ml)**
***24***	22.83 ± 0.24	16.31 ± 0.48	11 ± 8.3	5.7 ± 0.7	0	2 ± 0	0	0.3 ± 0.3	2.0 ± 0.6	0.43 ± 0.06
***36***	36.64 ± 0.83	16.80 ± 0.28	15.7 ± 5.0	8.3 ± 2.7	0.3 ± 0.3	0.3 ± 0.3	1.6 ± 0.3	1.0 ± 0.6	2.0 ± 0.6	0.54 ± 0.14
***48***	49.33 ± 2.16	13.10 ± 4.85	14.5 ± 2.5	6.0 ± 3.0	0.5 ± 0.5	0	2.5 ± 0.5	1.5 ± 1.5	1.5 ± 0.5	0.55 ± 0.13

Ewes were monitored through three estrous cycles then euthanized 24 (n = 3), 36 (n = 3), or 48 (n = 2) hours after the onset of the next estrus. Data are presented as means ± SEM. Ovarian structures were measured on the external surface of the ovary with a transparent ruler (to the nearest 0.5 mm) and recorded. All visible follicles were aspirated and follicular fluid from each pair of ovaries was pooled according to follicular diameter: small (≤ 2.0 mm), medium (2.1-4.0 mm), large (4.1-6.0 mm; gene expression in large follicles was not assessed due to low cell numbers) and preovulatory (≥ 6.1 mm). Blood was collected immediately prior to euthanasia.

Estrous cycle lengths of ewes were similar (Table 2) except for one ewe: Ewe 4 exhibited shorter cycles that averaged 8.25 days. Relative expression of FSHR variants within samples collected from Ewe 4 fell in the range of ewes with normal cycle length; as such, those results were included. The average number of follicles found per group are listed in Table 2.

Follicle totals (Table 2) were as follows: small (≤ 2.0 mm; n = 109 total; n = 8 pools), medium (2.1 – 4.0 mm; n = 54 total; n = 8 pools) large (4.1-6.0 mm; n = 1) and preovulatory (≥ 6.1 mm; n = 7 total; n = 3 pools). No large follicles were analyzed for RNA expression due to sampling errors and/or lack of GC recovery during aspiration. No significant differences were found between collection times (24, 36, or 48 hours after onset of estrus) for number of follicles within a size class.

## Discussion

We have shown that in untreated mature ewes allowed to cycle naturally, the relative expression of FSHR-3 was greater than FSHR-1 in cells collected from medium (2.1-4.0 mm) follicles and tended to be higher in cells collected from small follicles. These results provide the first evidence that FSHR-3 is not a rare transcript in sheep. Based on its structure, the FSHR-3 variant has been described to function as a growth factor type-I receptor for FSH through activation of the extracellular-regulated kinase (ERK) pathway in granulosa cells [10]. Regardless of follicle size, FSHR-3 expression appeared to exceed that of FSHR-1, indicating that FSHR-3 may be more important during follicular development in the ewe than previously thought. Expression of LHR was expected to increase with increasing granulosa cell differentiation (and follicle diameter) [16-18] and this was the case in the present experiment. Relative expression of FSHR-2 was the lowest of all FSHR variants, with expression only exceeding (although not significantly) that of FSHR-1 in preovulatory follicles.

The G protein-coupled form of the FSH receptor, FSHR-1 appeared to be expressed in follicles of all sizes at 24, 36, and 48 hours after the onset of estrus. These results agree with Tisdall et al. [1] who found FSHR to be expressed in follicles as small as the primary stage, with expression continuing thereafter, even in follicles beginning to undergo atresia [1]. However, Abdennebi et al. [16] and many others have attempted to assess FSHR expression using primers or probes that would not discriminate between the different FSHR variants, and thus it is not possible to determine which form of the FSHR was present, or to predict second messenger pathways used by FSH at different stages of follicular development. In the current study, care was taken to design primers such that specific variants of the FSHR would be discernable (Figure 1).

Other studies have been conducted that examined FSHR variant expression in both sheep and mice using RT-PCR and Western blotting. In one study, immature mice (21d) were PMSG- primed (5 IU) and ovaries were harvested 24 or 48 hours later [19]. A two-fold increase was seen in FSHR-3 expression over FSHR-1 in PMSG-primed mice; a corresponding increase in each receptor protein expression was also observed. McNatty et al. [20] showed that PMSG (500 IU) priming can decrease atresia of small antral follicles during luteolysis in sheep. These investigators also saw that at 24 hours, the proportion of healthy and atretic follicles was restored to pretreatment levels [20]. Thus, because the effects of PMSG are relatively short-lived, assessment of FSHR variant expression within follicles at 12 hours after treatment with PMSG should be considered in the future. In addition, dose–response experiments using PMSG in conjunction with measurement of FSHR variant expression would need to be performed before conclusions can be made to determine if PMSG stimulates alterations in FSHR variant expression in sheep.

To help determine the health of follicles collected in this experiment, progesterone and estradiol concentrations for follicular pools were examined. Steroid content of follicular fluid can be used as an indicator of follicular health status when looking at steroid profiles of individual follicles [21]. In the current study, follicles were pooled for each pair of ovaries based on size criteria, and accordingly, follicular health assessments based on estradiol and progesterone represented “average” follicle health and not actual health of each follicle in which FSHR variants were measured. Nevertheless, “average” follicle health may provide an initial indication of average FSHR variant expression based on stage of development.

Because follicles of similar sizes can have very different steroid profiles [22], assessing follicular health based on steroid content can be difficult. Results herein were similar to those of Somchit et al. [23], in that they observed higher progesterone than estradiol concentrations in pooled follicular fluid from follicles < 3.5 and ≥ 3.5 mm in diameter. We saw the same trend in pools of fluid from small (≤ 2.0 mm) or medium (2.1-4.0 mm) follicles. Because progesterone concentrations were consistently higher than estradiol, we believe that a greater number of atretic small or medium follicles were collected than healthy (growing) small or medium follicles. This is a reasonable assumption when considering the mechanism of follicular dynamics. Once follicles reach the preovulatory size, estradiol is no longer the primary hormone produced by the follicle [21], so parameters in addition to the E:P ratio are necessary when assessing follicular health of that follicle size class.

Serum progesterone was measured to help assess cycling status. Circulating progesterone concentrations during the luteal phase of ewes are about 3 ng/ml, while progesterone concentrations during estrus are generally less than 1 ng/ml [24]. For Experiment 1, progesterone concentrations collected just prior to euthanasia were under 1 ng/ml, so any corpora lutea observed when ovaries were examined after harvest were probably regressing. Low serum progesterone concentrations, taken together with estrous behavior observations, provide compelling evidence that detection of onset of estrus was accurate.

## Conclusions

The diverse actions of FSH on follicles during the estrous cycle are well documented, and include stimulation of differentiation, hormone production, and proliferation of granulosa cells. Some of these actions are mediated by FSHR-1 through activation of adenylate cyclase and production of cAMP. The G protein-coupled form of the receptor can also activate other signaling pathways, including extracellular regulated kinases (ERKs), and mitogen-activated protein kinases (MAPKs) [6]. The identification of the FSHR nucleotide sequence [2,5] made it possible to detect alternatively spliced variants of the FSHR [3,8,25]. The finding that FSHR-3 is the primary form of the FSHR in at least medium sized follicles examined in these experiments supports the idea that other forms of the FSHR, in addition to the well-characterized G protein-coupled form, may participate in follicular growth and development during the sheep estrous cycle.

## Abbreviations

FSH: Follicle stimulating hormone; FSHR: Follicle stimulating hormone receptor; FSHR-1: G protein-coupled FSHR; FSHR-2: Dominant negative FSHR; FSHR-3: Growth factor type I FSHR; HEK: Human embryonic kidney; PMSG: Pregnant mare serum gonadotropin.

## Competing interests

The authors declare that they have no competing interests.

## Authors’ contributions

RS carried out all aspects of the study, including work with ewes, tissue and blood collection, measurement and characterization of follicles and all assays, as well as preparing the manuscript. BF coordinated all activities with animals, placed and removed CIDRs, took blood samples and helped plan the design of all experiments. DE helped to validate and perform all molecular assays, including reverse transcription and real time PCR. DG helped with experimental design, validation of all assays and laboratory procedures, and critically evaluated the manuscript. AC performed and coordinated tissue collection and evaluated ewe health and welfare status, and critically evaluated the manuscript. TR conceived of the project, coordinated all animal, tissue collection and laboratory activities and performed final edits of the manuscript. All authors read and approved the final manuscript.
